# Persistence of accuracy of genome-wide breeding values over generations when including a polygenic effect

**DOI:** 10.1186/1297-9686-41-53

**Published:** 2009-12-29

**Authors:** Trygve R Solberg, Anna K Sonesson, John A Woolliams, Jørgen Ødegard, Theo HE Meuwissen

**Affiliations:** 1 Norwegian University of Life Sciences, Department of Animal and Aquacultural Sciences, PO Box 5003, N-1432 Ås, Norway; 2 NOFIMA Marine, PO Box 5010, N-1432 Ås, Norway; 3 Roslin Institute (Edinburgh), Roslin, Midlothian EH25 9PS, UK

## Abstract

**Background:**

When estimating marker effects in genomic selection, estimates of marker effects may simply act as a proxy for pedigree, i.e. their effect may partially be attributed to their association with superior parents and not be linked to any causative QTL. Hence, these markers mainly explain polygenic effects rather than QTL effects. However, if a polygenic effect is included in a Bayesian model, it is expected that the estimated effect of these markers will be more persistent over generations without having to re-estimate the marker effects every generation and will result in increased accuracy and reduced bias.

**Methods:**

Genomic selection using the Bayesian method, 'BayesB' was evaluated for different marker densities when a polygenic effect is included (GWpEBV) and not included (GWEBV) in the model. Linkage disequilibrium and a mutation drift balance were obtained by simulating a population with a Ne of 100 over 1,000 generations.

**Results:**

Accuracy of selection was slightly higher for the model including a polygenic effect than for the model not including a polygenic effect whatever the marker density. The accuracy decreased in later generations, and this reduction was stronger for lower marker densities. However, no significant difference in accuracy was observed between the two models. The linear regression of TBV on GWEBV and GWpEBV was used as a measure of bias. The regression coefficient was more stable over generations when a polygenic effect was included in the model, and was always between 0.98 and 1.00 for the highest marker density. The regression coefficient decreased more quickly with decreasing marker density.

**Conclusions:**

Including a polygenic effect had no impact on the selection accuracy, but showed reduced bias, which is especially important when estimates of genome-wide markers are used to estimate breeding values over more than one generation.

## Background

High-throughput genotyping and availability of dense marker information have made prediction of breeding values based on dense marker genotyping possible resulting in so-called genome-wide breeding values (GWEBV). Several methods have been suggested to estimate marker effects in the prediction of GWEBV e.g. [1-4]. The advantage of selecting parents on GWEBV in genomic selection schemes is the potential to select candidates with high accuracy and low bias directly by using marker genotypes or haplotypes only. Several simulation studies in which markers were calibrated on a training set of phenotypes e.g. [5-8] have demonstrated these advantages. GWEBV may reduce the amount of phenotyping required for breeding schemes and hence constitute an attractive proposition because obtaining phenotypic records routinely for all generations can be expensive, reduce animal welfare, and sometimes impossible for live selection candidates. An example for which all three issues apply is in fish aquaculture where selecting disease resistance is done by challenging sibs of the candidates with the disease to avoid infecting the selection candidates [9]. Genomic selection selects animals directly on the genotype, rather than the phenotype which may be an advantage, especially for traits that cannot be measured or are expensive to record on selection candidates (e.g. slaughter traits and challenge-test data).

Successful implementation of genomic selection relies on some underlying assumptions. One assumption is the existence of population-wide linkage disequilibrium (LD) between markers and quantitative trait loci (QTL). As a result of imperfect LD, markers in LD with the QTL are not likely to explain all existing genetic variation, and the remaining genetic variation will be included in the polygenic variance. For sparse marker maps, linkage disequilibrium between the markers and the QTL will be reduced and only part of the genetic variance will be explained by the markers. Furthermore, estimated marker effects may model family relationships [6], which will result in spurious associations between phenotypes and marker alleles, i.e. there will be non-zero marker effects whilst the marker alleles are not linked to any causative QTL. It is expected that such marker-QTL associations decay at a rate of (1-*c*)^*t*^, where *c *is the distance between marker and QTL and *t *is the number of generations [10]. For spurious associations, *c *= 0.5, and for tightly linked markers, *c *< 0.01. Thus, spurious associations decay much more rapidly than associations based on real linkage over time. This introduces the important issue of the persistence of GWEBV predictions over generations in the absence of marker effects re-estimation, and few studies have examined this issue, e.g. [11].

One solution that may address both issues is to include a polygenic effect in the model and this has been addressed by others, e.g. [6,7,12], but not evaluated over multiple generations. Our hypothesis is that these spurious associations are better represented by a polygenic effect in the model than by markers that happen to have a higher frequency in some families compared to others. Thus it is expected that including a polygenic effect will capture genetic variation that is not in tight linkage with markers, such that this variation is to a lesser extent captured by markers through spurious associations. The objective of this study was to test this hypothesis by investigating the effect of including a polygenic effect into a Bayesian model for the estimation of marker estimates to predict GWEBV, and their accuracy and bias over multiple generations to study their persistence over time.

## Methods

### Population structure and genome size

Details of the simulation model have been described in an earlier paper [8]. Briefly, a population with an effective population size of Ne = 100 was simulated over 1000 generations of random mating, random selection and with a genome subject to mutation. In generation *t *= 1001, the number of animals was increased to 1000 by factorial mating of 50 sires (i = 1-50) and 50 dams (i = 51-100) from generation *t *= 1000. The factorial mating was achieved by mating sire 1 to dams 51-70, sire 2 to dams 52-71, sire 3 to dams 53-72 and so on, and each dam had one offspring per sire. In descending generations (*t *= 1002 to *t *= 1006), the animals had 1000 offspring produced by random sampling with replacement among the parents selected from the previous generation.

The size and structure of the genome were the same as described in [8]. The genome (10 Morgan) was simulated with 10 chromosomes each 100 cM long. Four density schemes were evaluated, and the density was scaled by the effective population size (Ne) used to generate the markers, which was Ne = 100 and a genome size in Morgan (M). Scaled marker densities were 1, 2, 4 and 8 Ne/M, which corresponded here to 100, 200, 400 and 800 markers per Morgan.

Mendelian inheritance and the Haldane mapping function were assumed for all loci. The mutation rate of the markers was assumed to be 2.5 × 10^-3 ^per locus per meiosis. With this mutation rate, 99% of the potential markers were segregating at *t *= 1001. Markers with more than two alleles segregating at *t *= 1001 were converted to bi-allelic SNP markers by ignoring some of the mutations as described in [8]. The minor allele frequencies of the SNP markers showed approximately a uniform distribution with an over-representation of marker alleles with intermediate frequencies, which in practice may reflect the effect of pre-screening SNP markers and selecting the most informative. The potential number of QTL was kept at 100 per chromosome, distributed evenly over each chromosome. The actual number of segregating QTL at *t *= 1001 depended on the mutation rate which was assumed to be 2.5 × 10^-5 ^per locus per meiosis. The resulting number of segregating QTL was typically 5 to 6% of the potential number. The additive effect of a mutational allele of the multi-allelic QTL was sampled from the gamma distribution with a shape parameter of 0.4 and scale parameter of 1.66 [13] with an equal probability of a positive or negative effect. No polygenic effect was simulated.

### True breeding value (TBV) and phenotypic values

The true breeding value (TBV) of animal *i *from generation 1001-1006 was calculated as:

where **q**_j _is a vector of true QTL effects of the QTL alleles at locus *j*, and **Q**_ij _is an incidence row vector indicating for animal *i *which of the QTL alleles it carried at locus *j *(e.g. Q_ij _= [1 1 0 ..]' for animal *i *carrying QTL alleles 1 and 2 at locus *j*); N_QTL _is the number of QTL loci. For generation 1001, phenotypic values for each animal were simulated as: y_i _= TBV_i _+ ε_i_, where ε_i _~ N(0, σ^2^_e_). The variance of the TBV effects (σ^2^_TBV_) varied somewhat from replicate to replicate, but was on average 1.0 (s.e. = 0.118). The environmental variance (σ^2^_e_) was set equal to σ^2^_TBV _such that the heritability was 0.5 for every replicate.

### Estimation model with polygenic effect

The 'BayesB' method of Meuwissen *et al*. [3] was used to estimate the effects of the SNP markers for the 1000 animals in generation *t *= 1001. The 'BayesB' model is described in more detail in earlier papers [3] and [8], but briefly, the variance of the marker effects (σ^2^_gi_) was estimated for every marker using a relevant prior distribution which was a mixture of an inverted chi-squared distribution and a discrete probability mass at σ^2^_gi _= 0. A Metropolis-Hastings algorithm was used to sample σ^2^_gi _from its distribution conditional on y*, *p*(σ^2^_gi _| y*), where y* denotes the data **y **corrected for the mean and all other genetic effects except the marker effect (**g**_i_) [14]. Given σ^2^_gi_, marker effects, **g**_i _were sampled from a Normal distribution as prior and using Gibbs sampling [15]. The 'BayesB' model was extended to include a polygenic effect (**a**):

where **y **is the vector of phenotypes, μ is the overall mean, **a **is the vector of polygenic effects,  is the summation over all marker loci from 1 to *Nloc*, where *Nloc *is varying from 1010 marker loci for the lowest marker density (1Ne/M) to 8080 marker loci for the highest marker density (8Ne/M). **X**_j _is a design matrix for the *j*'th marker, **g**_j _is the vector of the *j*'th marker effect and **e **is the residual term. Dimension of the **y, a**, and **e **vectors are 1000 × 1, the **X**_j _matrix varies from 1010 × 2 for the lowest marker density up to 8080 × 2 for the highest marker density. The variance of **a **was Var(**a**) = **A**σ^2^_a_, where **A **(1000 × 1000) is the additive relationship matrix, calculated based on five generations of pedigree from generation *t *= 996 to *t *= 1000 using the algorithm of [16]. Polygenic effects were sampled in the MCMC chain using Gibbs sampling and assuming a prior N(0, σ^2^_a_) following [15], and σ^2^_a _was estimated using a scaled inverted chi-squared prior distribution with -2 degrees of freedom, which implies a non-informative flat prior distribution [15].

The variance of the marker effect was σ^2^_gj_, which was estimated for every marker using a mixture distribution as the prior;

The probability *p *depends on the density of the markers, and varies with different marker densities, because with more markers, it becomes less likely for marker *j *to be required to capture the predictive LD between QTL and markers, i.e. *p *= 53/(Nloc) where 53 is the expected number of QTL and Nloc is the number of marker loci. Sampling from the posterior distribution of σ^2^_gj_, was by a Metropolis-Hastings algorithm that sampled σ^2^_gj _from *p*(σ^2^_gj _| y*), where the prior distribution, *p*(σ^2^_gj_), was used as the distribution to suggest updates for the Metropolis Hastings chain [13], and y* denotes the data **y **corrected for the mean and all other genetic effects except the marker effect (**g**_j_). The Metropolis Hastings chain was run for 10.000 cycles using a burn-in period of 1000 cycles. Given σ^2^_gj_, marker effects, **g**_j _were sampled from *p*(g_j _| σ^2^_gj_) using Gibbs sampling [15].

### Prediction of genome-wide breeding values

Prediction of the GWEBV for the method 'BayesB' without polygenic effect was calculated from:

where **X**_ij _denotes the marker genotype of animal *i *at locus *j *in generation *t *= 1002 to *t *= 1006, and  is the estimate of the marker effects, which was estimated on animals in generation *t *= 1001.

Prediction of the breeding values including the polygenic effect (GWpEBV) for the method 'BayesB' was calculated from:

Since no data was recorded after generation *t *= 1001, the polygenic effect  of animal *i *in generation *t *was calculated as  where the subscripts *s *and *d *represent the sire and dam of animal *i*, respectively. This formula is valid here because the parents of the next generation were randomly selected and there was no phenotypic data entering the evaluations in later generations, i.e. after generation *t *= 1001. For each replicate, the mean and median of the Gibbs samples for the polygenic variance were calculated from the final 5000 values of the chain. These values were then averaged over 10 replicates.

As a measure of bias we calculated the linear regression coefficient of true breeding values on GWEBV and GWpEBV within each of the five generations from *t *= 1002 to *t *= 1006. The correlation coefficients were calculated between the true breeding value and the GWEBV and GWpEBV for all five generations which reflects the accuracy of predicting the genome-wide breeding values. The result is based on the average of 20 replicates for each marker density.

## Results

### Accuracy of selection

Tables 1 to 4 show the accuracy of selection and bias for the four marker densities when the polygenic effect (GWEBV) is ignored and when it is included (GWpEBV). For the highest marker density (8Ne/M), the selection accuracy decreased from 0.875 in generation *t *= 1002 to 0.842 in generation *t *= 1006 for GWEBV (Table 1). Selection accuracy was higher for GWpEBV than for GWEBV for all generations and this was particularly significant for three out of five generations. The difference in accuracy between the two models varied from 0.008 in generation *t *= 1002 to 0.014 in generation *t *= 1006. The decrease in accuracy from one generation to the next was similar for the two models.

**Table 1:** Selection accuracy (r) and regression of TBV on GWEBV and GWpEBV over five generations for marker density 8Ne/M, and accuracy differences when a polygenic effect is included

	Accuracy of selection		Regression of TBV on GWEBV	
**Generation**	**r_GWEBV_^i) ^± s.e**	**Δ_r_^ii) ^± s.e**	**b_GWEBV_^i) ^± s.e**	**Δ_b_^ii) ^± s.e**

*t *= 1002	**0.875** 0.006	**0.008** 0.003	**0.926** 0.008	**0.058** 0.012

*t *= 1003	**0.861** 0.007	**0.007** 0.005	**0.917** 0.010	**0.068** 0.014

*t *= 1004	**0.857** 0.007	**0.007** 0.006	**0.916** 0.010	**0.074** 0.013

*t *= 1005	**0.852** 0.008	**0.011** 0.005	**0.912** 0.012	**0.080** 0.011

*t *= 1006	**0.842** 0.009	**0.014** 0.006	**0.906** 0.011	**0.079** 0.010

^i)^GWEBV represents the genome-wide estimated breeding value without polygenes

^ii)^Δ represents the difference between genome-wide estimated breeding values including a polygenic effect (GWpEBV) and GWEBV (Δ_r _= r_GWpEBV _- r_GWEBV _and Δ_b _= b_GWpEBV _- b_GWEBV_)

**Table 2:** Selection accuracy (r) and regression of TBV on GWEBV and GWpEBV over five generations for marker density 4Ne/M and accuracy differences when a polygenic effect is included

	Accuracy of selection		Regression of TBV on GWEBV	
**Generation**	**r_GWEBV_^i) ^± s.e**	**Δ_r_^ii) ^± s.e**	**b_GWEBV_^i) ^± s.e**	**Δ_b_^ii) ^± s.e**

*t *= 1002	**0.795** 0.006	**-0.014** 0.003	**0.896** 0.010	**-0.027** 0.009

*t *= 1003	**0.756** 0.006	**-0.002** 0.007	**0.864** 0.011	**0.032** 0.015

*t *= 1004	**0.732** 0.007	**0.006** 0.007	**0.848** 0.011	**0.065** 0.013

*t *= 1005	**0.722** 0.007	**0.011** 0.006	**0.846** 0.010	**0.075** 0.012

*t *= 1006	**0.705** 0.008	**0.014** 0.007	**0.827** 0.010	**0.095** 0.011

^i)^GWEBV represents the genome-wide estimated breeding value without polygenes

^ii)^Δ represents the difference between genome-wide estimated breeding values including a polygenic effect (GWpEBV) and GWEBV (Δ_r _= r_GWpEBV _- r_GWEBV _and Δ_b _= b_GWpEBV _- b_GWEBV_)

**Table 3:** Selection accuracy (r) and regression of TBV on GWEBV and GWpEBV over five generations for marker density 2Ne/M and accuracy differences when a polygenic effect is included

	Accuracy of selection		Regression of TBV on GWEBV	
**Generation**	**r_GWEBV_^i) ^± s.e**	**Δ_r_^ii) ^± s.e**	**b_GWEBV_^i) ^± s.e**	**Δ_b_^ii) ^± s.e**

*t *= 1002	**0.801** 0.008	**-0.014** 0.006	**0.889** 0.011	**-0.074** 0.014

*t *= 1003	**0.763** 0.010	**0.007** 0.008	**0.862** 0.011	**-0.001** 0.012

*t *= 1004	**0.736** 0.011	**0.026** 0.006	**0.837** 0.013	**0.048** 0.014

*t *= 1005	**0.722** 0.011	**0.036** 0.008	**0.814** 0.012	**0.106** 0.009

*t *= 1006	**0.717** 0.009	**0.036** 0.007	**0.811** 0.010	**0.104** 0.010

^i)^GWEBV represents the genome-wide estimated breeding value without polygenes

^ii)^Δ represents the difference between genome-wide estimated breeding values including a polygenic effect (GWpEBV) and GWEBV (Δ_r _= r_GWpEBV _- r_GWEBV _and Δ_b _= b_GWpEBV _- b_GWEBV_)

**Table 4:** Selection accuracy (r) and regression of TBV on GWEBV and GWpEBV (b) over five generations for marker density 1Ne/M and the accuracy differences when a polygenic effect is included

	Accuracy of selection		Regression of TBV on GWEBV	
**Generation**	**r_GWEBV_^i) ^± s.e**	**Δ_r_^ii) ^± s.e**	**b_GWEBV_^i) ^± s.e**	**Δ_b_^ii) ^± s.e**

*t *= 1002	**0.679** 0.006	**0.005** 0.008	**0.866** 0.014	**-0.016** 0.013

*t *= 1003	**0.610** 0.009	**0.011** 0.012	**0.794** 0.017	**0.056** 0.026

*t *= 1004	**0.565** 0.013	**0.010** 0.013	**0.732** 0.015	**0.088** 0.023

*t *= 1005	**0.535** 0.013	**0.007** 0.015	**0.701** 0.016	**0.089** 0.024

*t *= 1006	**0.518** 0.012	**0.013** 0.013	**0.684** 0.016	**0.101** 0.025

^i)^GWEBV represents the genome-wide estimated breeding value without polygenes

^ii)^Δ represents the difference between genome-wide estimated breeding values including a polygenic effect (GWpEBV) and GWEBV (Δ_r _= r_GWpEBV _- r_GWEBV _and Δ_b _= b_GWpEBV _- b_GWEBV_)

For both intermediate marker densities, the accuracy of GWpEBV in generation *t *= 1002 was lower compared to that of GWEBV (p < 0.05), but after five generations of selection, the accuracy was significantly higher for GWpEBV (Tables 2 and 3) (p < 0.05). For example, for marker density 4Ne/M, the difference in accuracy between GWpEBV and GWEBV was -0.014 in generation *t *= 1002 and 0.014 after five generations (Table 2), indicating that by including a polygenic effect retained greater accuracy over generations. The accuracies for marker density 2 Ne/M are relatively high compared to those for higher marker densities, which may be due to the structure of the marker/QTL map. At a marker density of 2 Ne/M, every SNP is adjacent to a putative QTL, whereas at higher densities a fraction of the SNP is not adjacent to any QTL [13].

For the lowest marker density (1N_e_/M), the accuracy was 0.679 in the first generation for GWEBV and reduced to 0.518 in generation *t *= 1006 (Table 4). The use of GWpEBV increased the accuracy from 0.005 in generation *t *= 1002 to 0.013 in generation *t *= 1006.

**Figure 1:**
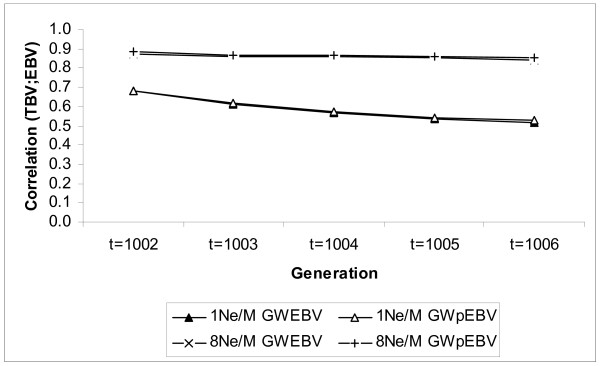
**Accuracy of selection over five generations in different models**. Selection accuracy was determined for marker densities of 1Ne/M and 8Ne/M with the polygenic effect included (GWpEBV) or not (GWEBV) in the model; the lines for GWEBV and GWpEBV overlap almost completely, indicating minor differences between the two models.

In general, when the polygenic effect was included in the model, the accuracy of GWpEBV was reduced in later generations as for GWEBV, but the decrease in accuracy was smaller for GWpEBV, especially for the intermediate marker densities. Figure 1 illustrates the selection accuracy over five generations for GWpEBV and GWEBV for the highest and lowest marker densities, and the difference in accuracy between the two marker densities increased as the number of generations increased. Figure 1 clearly shows marginal differences between GWpEBV and GWEBV for the two marker densities, since the lines overlap, and that the accuracy is more stable over generations using a high marker density compared to a low marker density.

### Regression coefficient of TBV on GWEBV and GWpEBV

The linear regression coefficient of TBV on GWEBV and GWpEBV was used as a measure of bias for these two selection criteria. Table 1, 2, 3, 4 show the regression coefficients of TBV on GWEBV and GWpEBV and the difference between the two models. For the highest marker density (8Ne/M), the regression coefficient for GWEBV was 0.926 in generation *t *= 1002 and reduced to 0.902 in generation *t *= 1006 (Table 1). The regression coefficient was significantly higher for GWpEBV than for GWEBV for all generations, and the difference between the models varied from 0.058 in generation *t *= 1002 to 0.079 in generation *t *= 1006, respectively. Consequently the regression coefficients for GWpEBV were always between 0.98 and 1.00, i.e. showing only a very small bias. The reduction in regression was larger for GWEBV than for GWpEBV, as the regression coefficient for GWpEBV was much more stable over generations.

For the intermediate marker densities, the regression coefficients were lower. However, there was a marked interaction between generation and method. For GWpEBV the regression coefficient was smaller than that for GWEBV at generation *t *= 1002, but increased slightly over generations. In contrast, the regression coefficients for GWEBV decreased steadily over generations (Tables 2 and 3). By generation *t *= 1006, the difference in regression coefficients between GWEBV and GWpEBV was substantial: 0.095 (s.e. = 0.011) and 0.104 (s.e. = 0.010) for 4Ne/M and 2Ne/M, respectively. For 1Ne/M, both methods showed the same trend i.e. a decreasing regression coefficient, but the rate of decrease was faster for GWEBV.

**Figure 2:**
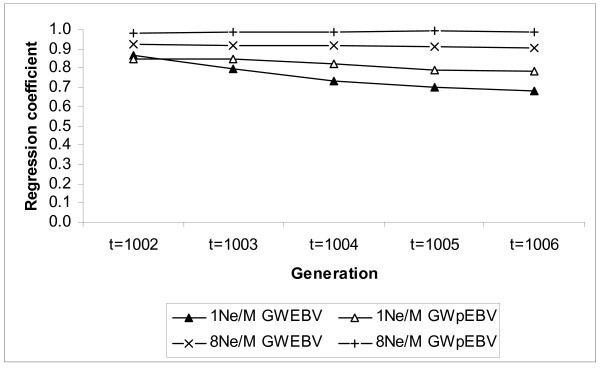
**Regression coefficients of TBV on GWEBV and GWpEBV (bias) over five generations for marker densities 1Ne/M and 8Ne/M**.

In general, if the polygenic effect was ignored, bias increased from generation *t *= 1002 to *t *= 1006 for all four marker densities. However, this bias decreased with increasing marker densities (Table 1, 2, 3, 4). If a polygenic effect was included, the situation was similar, but the bias for all marker densities was more stable over generations, and furthermore decreased for intermediate generations (Table 2 and 3). For marker density 8Ne/M, the regression coefficient remained between 0.98 and 1.00 for all generations. Figure 2 shows the regression coefficient of TBV on GWEBV and GWpEBV for the highest marker density compared to the lowest marker density, and clearly shows that the regression coefficient is more stable over generations when the marker density is high and a polygenic effect is included.

### Polygenic variance

Table 5 shows the mean and median of the polygenic variances for the four different marker densities. The estimate of the mean polygenic variance ranged from 0.267 for 1Ne/M to 0.411 for 8 Ne/M with large standard errors. There was no statistically significant difference between the different marker densities, and no statistical evidence of a trend with increasing marker density. The distribution of the values for the Gibbs sampling within a replicate was very large with sporadic extreme values. This prompted us to examine the medians for the Gibbs samples for each replicate, they may be more robust to such outliers; however the picture changed very little.

**Table 5:** Mean and median polygenic variance for the different marker densities in the base generation (*t *= 996), estimated from the analysis of phenotypes in generation *t *= 1001

	1Ne/M	2Ne/M	4Ne/M	8Ne/M
Mean (s.e.)	**0.267** (0.090)	**0.323** (0.056)	**0.360** (0.028)	**0.411** (0.070)

Median (s.e.)	**0.266** (0.099)	**0.272** (0.059)	**0.252** (0.022)	**0.403** (0.082)

## Discussion

This study shows that including a polygenic effect has little impact on the accuracy of genome-wide EBVs in the generation immediately following phenotyping. However, as the generations progress, the predictions with the polygenic effect retains somewhat greater accuracy. This persistence in accuracy over time is particularly significant for higher marker densities. This is because spurious marker associations arising from the pedigree are reduced, so that the remaining marker associations reflect more truly LD through proximity on the chromosome, which changes more slowly over time. Likewise the bias of the GWpEBV is significantly reduced compared to GWEBV, and the reduction is larger for the lowest marker densities, which displayed the largest bias for GWEBV. With lower marker densities, there are fewer markers around the QTL to explain the effect of the QTL, and the polygenic variance is expected to be more important for providing information for the estimated breeding values.

In Calus *et al*. [12], the accuracy of genomic selection including a polygenic effect was related to linkage disequilibrium (LD) between adjacent markers. For a high heritability trait, they found that including a polygenic effect increased selection accuracy when the r^2 ^was lower than 0.14, and the benefit of including a polygenic effect increased with reduced r^2^. The latter is consistent with our results in generation *t *= 1002, which is the only generation that can be compared to this study. In Calus *et al*. [12], the simulated model was based on a lower number of markers, smaller genome size and did not study the ability to predict GWEBV over multiple generations. The r^2 ^values were calculated for a very similar dataset in an earlier paper, and were between 0.16 and 0.35 [8], which are larger than what Calus *et al*. [12] reported. As found here, the advantage of including a polygenic effect is more limited in the first generation after estimating marker effects. However, in practical situations, it may be advantageous to estimate the marker effects in one generation (e.g., due to phenotypic costs), and use these effects to select animals over multiple generations. Under such circumstances, it would be advantageous to include a polygenic effect since the accuracy will increase and bias decrease.

Whilst the accuracy of selection is a primary parameter of interest in animal breeding, the bias is also relevant since it determines the model's ability to predict the genetic progress. When generations are overlapping, individuals with different amounts of information and genetic level need to be compared for selection and biases, which can reduce the accuracies in predicting breeding values. Our results indicate that the polygenic effect did account for some of the variance not captured by the markers. Since estimates of polygenic effects are based on the BLUP theory and will thus show small bias, it may be expected and was found that including a polygenic effect reduces the bias.

The estimates of the variance of the polygenic effect increase with increasing marker density (Table 5), which is contrary to our expectation that, as marker density increases, the QTL will be more closely modelled by the markers and polygenic effects will become less important. A possible explanation for this result is that the non-linear regression implied by BayesB estimation of marker effects becomes more non-linear as marker density increases (because the fraction of markers with non-zero effect is expected to decrease). The increased non-linearity of the regression implies that small spurious associations will be increasingly regressed back to zero, resulting in more variance being explained by the polygenic effect. Furthermore, on a per marker basis, the spurious associations become smaller, since they are spread over more markers. These reductions in marker effects due to spurious associations may result in an increased variance attributed to the polygenic effect. This explanation implies that the effect of including or excluding a polygenic effect in these Bayesian models may depend on the prior distributions used for the marker effects and the polygenic effect, and different prior distributions may result in different outcomes. Also the number of QTL simulated (50-60) may affect the importance of the polygenic effect. It may be expected that with more QTL, the genetic model will become more like the infinitesimal model and the inclusion of a polygenic effect may be more beneficial.

Depending on the cost of genotyping and numbers of markers used, genomic selection programs will be more cost effective if the estimated marker effects could be used over multiple generations. Recombination will occur between the markers and QTL over time, resulting in reduced r^2 ^and reduction in the accuracy of selection. This study shows that a marker density of 8Ne/M seems sufficient for the estimated marker effects to persist over five generations with minimum bias and only a small reduction in selection accuracy. However, in practice the results will depend on the genetic architecture of the genome and on how similar the simulated parameters used in the study are compared to real genomes. Nevertheless, including a polygenic effect is beneficial for a random mating population when estimated marker effects are used to predict GWEBV over multiple generations, especially with respect to the bias.

## Competing interests

The authors declare that they have no competing interests.

## Authors' contributions

TRS simulated the datasets, carried out the analysis and drafted the manuscript. THEM wrote the computer modules and helped to carry out the study and draft the manuscript. All authors have read and approved the final manuscript.
